# Evaluation of risk factors for thromboembolic events in multiple myeloma patients using multiple machine learning models

**DOI:** 10.1097/MD.0000000000041428

**Published:** 2025-02-14

**Authors:** Yi Huang, Haimei Liang, Shaoxin Huang, Xueli Xie, Bin Deng, Wenjie Liang

**Affiliations:** a Department of Hematology, Guigang City People’s Hospital, Guigang, Guangxi, China.

**Keywords:** gradient hoist model, logistic regression model, multiple myeloma, random forest model, venous thromboses events

## Abstract

Venous thromboembolic events (VTE) is a frequent complication in multiple myeloma (MM) patients, raising mortality. This study aims to use machine learning to identify VTE risk factors in MM, helping to pinpoint high-risk individuals for better clinical management and prognosis. A retrospective analysis was conducted on the basic information, laboratory test results, treatment plans, and thrombosis prevention measures of 428 newly diagnosed MM patients at our hospital from December 2018 to December 2022. We used logistic regression (LR), random forest, and gradient boosting machine (GBM) models to identify and assess the risk factors for VTE in patients with MM. Among 428 patients with MM, 48 cases (11.21%) had concomitant VTE, including 10 cases of deep vein thrombosis with pulmonary embolism, while the remaining 38 cases were solely deep vein thrombosis. The results of the multifactorial LR analysis indicate that C-reactive protein (CRP), fibrinogen, von Willebrand factor (vWF), factor VIII (FVIII), and treatment regimen immunomodulator in the patient’s treatment regimen are independent factors influencing the risk of VTE in patients with MM. In the analysis of the random forest model, we found that CRP and fibrinogen are the most important factors for predicting the risk of VTE in patients with MM, with the highest Gini indices of 12.76 and 12.31, respectively. In addition, vWF, FVIII, age, platelet count, D-dimer, β2 microglobulin, serum creatinine, and albumin were also considered key variables affecting the risk of VTE in MM patients. In the GBM model, the importance ranking of variables showed that FIB and CRP are the most important predictive factors, with influences of 36.84 and 28.56, respectively. In addition, other important variables include vWF, FVIII, age, albumin, neutrophils, β2 microglobulin, and D-dimer. We found that CRP and fibrinogen were the most important risk factors in all 3 models, while vWF and FVIII were also confirmed as significant risk factors. The identification of these common risk factors provides a clear focus for clinical practice to more accurately identify high-risk groups for VTE among MM patients.

## 1. Introduction

Multiple myeloma (MM) is a common hematological malignancy characterized by abnormal proliferation of malignant plasma cells in the bone marrow and destructive bone damage.^[1]^ The annual incidence of MM in China is about 2 to 3 per 100,000, and most of the patients are middle-aged and elderly.^[2]^ Despite advances in medical technology and the ongoing application of various new drugs and therapies, which to some extent has improved the disease progression of MM patients, they still face many risks of complications, with the most common and serious being venous thrombotic events (VTE).^[3]^ VTE can lead to delays or interruptions in cancer treatment, affecting disease control and treatment effectiveness. It can also result in limited physical activity, pain, fatigue, difficulty breathing, and even a reduced overall survival rate.^[4]^

The National Comprehensive Cancer Network guidelines have proposed risk stratification for thrombosis events in MM and recommended prophylactic anticoagulation therapy for high-risk individuals.^[5,6]^ However, clinical trials and relevant literature have shown that these guidelines fail to clearly distinguish high-risk individuals. Therefore, the research team developed 2 clinical risk assessment models, namely the IMPEDE VTE score^[7]^ and the SAVED score,^[8]^ based on these guidelines in an attempt to improve risk stratification. However, additional biomarkers are still needed to enhance clinical effectiveness.

Identifying the risk factors for VTE in MM patients is crucial for timely anticoagulation measures and improving patient outcomes. Therefore, to effectively identify the risk factors for thrombus formation, we retrospectively collected demographic data, clinical characteristics, and serum biochemical indicators of MM patients. We utilized logistic regression (LR), random forest (RF), and gradient boosting machine (GBM) models to comprehensively analyze the impact of different clinical features on the risk of VTE in MM patients.

## 2. Objects and methods

### 2.1. Ethical approval

The Ethics Committee of Guigang City People’s Hospital approved this study (reference number: GYLLPJ-20221104-86), and all patients were asked to sign a written informed consent.

### 2.2. Subjects

The clinical data of MM patients newly diagnosed in our hospital from December 2018 to December 2022 were retrospectively analyzed.

Inclusion criteria: patients who meet the diagnostic criteria for MM as defined by the International myeloma working group.^[9]^ Age ≥18 years, any gender. Patients who have not received any cancer treatment prior to admission. Patients with relatively complete clinical data.

Exclusion criteria: patients with solitary plasmacytoma, smoldering myeloma, or plasma cell leukemia. Patients with concomitant severe heart, liver, lung, or brain diseases, or second malignancies. Patients who were previously treated at another hospital and transferred to our hospital during the course of treatment. Patients with a history of congenital coagulation disorders or long-term oral antiplatelet or anticoagulant therapy (such as aspirin and warfarin) due to conditions like cardiac stent implantation. Breastfeeding and pregnant women. Follow-up time <6 months.

Four hundred twenty-eight MM patients were included in this study. Based on the rule of having at least ten events per variable, the reported incidence of thromboses is approximately 10%,^[10,11]^ suggesting that the data is sufficient to establish a model.^[12]^ The patients were divided into the VTE group and the non-VTE group based on the occurrence of VTE events.

### 2.3. Grouping and diagnosis

Based on the occurrence of VTE events, patients were divided into the VTE group and the non-VTE group. VTE events primarily included deep vein thrombosis and pulmonary embolism. Deep vein thrombosis was diagnosed through vascular ultrasound, computed tomography venography, or angiography. pulmonary embolism was diagnosed using computed tomography pulmonary angiography, nuclear ventilation/perfusion imaging, magnetic resonance pulmonary angiography, and pulmonary angiography.

### 2.4. Collection of data

(1) Basic information: age, gender, body mass index, disease classification, Durie–Salmon (DS) staging, International staging system (ISS) staging, presence of pathological fractures, central venous catheter (CVC), etc.
(2) Laboratory test results: platelet count, neutrophil count, serum albumin, white blood cell, albumin, β2-microglobulin, C-reactive protein (CRP), lactate dehydrogenase, serum creatinine, fibrinogen, D-dimer, von Willebrand factor (vWF), coagulation factor VIII (FVIII), etc. All laboratory parameters were collected from patients at their initial visit, prior to any specific treatment for MM.
(3) Thrombosis status: treatment regimen and thrombosis prevention.

### 2.5. Statistical analysis

We used SPSS 23.0 (Chicago) for statistical analysis. For continuous data, such as age and laboratory indicators, if the data followed a normal distribution, we described it using mean ± standard deviation, and applied *t*-tests or nonparametric tests for verification. For categorical data, such as gender, DS stage, and ISS stage, we expressed it using frequency and percentage (n%). The statistical test used for this data was the chi-square test. *P* < .05 indicates that the difference is statistically significant.

We used R 4.2.1 software to construct LR, RF, and GBM models to comprehensively analyze the risk factors for VTE in MM. Through the LR model, we were able to quantify each factor’s relative impact on thrombotic risk and generate corresponding OR. The RF model improved the identification of thrombotic risk factors by constructing multiple decision trees and integrating the predictions from each tree. We also calculated the importance scores of each variable to determine the most influential factors in predicting thrombotic risk. GBM is suitable for handling different types of data and effectively identifies potential risk factors and their interactions. By comparing and combining these 3 models, we aim to provide a comprehensive perspective that reveals the key risk factors for thrombotic risk in patients with MM.

## 3. Results

### 3.1. General information of the subjects

Finally, 428 patients who met the requirements of the study were included in this study, including 48 cases (11.21%) of VTE and 380 cases (88.79%) of non-VTE. 48 patients with VTE were mainly deep vein thrombosis, 10 cases of lower extremity deep vein thrombosis combined with pulmonary embolism, and the remaining 38 cases were simple deep vein thrombosis. 28 cases of thrombotic events occurred within 6 months after diagnosis, 9 cases of VTE occurred after 7 to 12 months, and 11 cases of VTE occurred after 12 months. After randomly splitting the dataset in an 80:20 ratio, there were 342 cases in the training set and 86 cases in the validation set.

### 3.2. Univariate analysis of VTE in MM patients

The patients in the training set were divided into VTE group (31 cases) and non-VTE group (311 cases). Univariate analysis was performed to screen potential influencing factors with *P* < .05 as the threshold. The results showed that age, CVC, CRP, fibrinogen, D-dimer, vWF, FVIII, and treatment regimen immunomodulator (IMiD) were potential influencing factors for VTE in MM patients (see Table 1).

**Table 1:** Univariate analysis of VTE in MM patients.

Variables	VTE (n = 31)	non-VTE (n = 311)	t/*x*^2^	*P*
Age (yr) (mean, SD)	51.23 ± 6.87	56.84 ± 6.05	4.377	<.001
Body mass index (kg/m^2^) (mean, SD)	22.74 ± 2.78	23.19 ± 2.70	0.888	.375
Gender (n, %)				
Female	12 (38.71)	147 (47.27)	0.830	.362
Male	19 (61.29)	164 (52.73)		
DS (n, %)				
<III	7 (22.58)	100 (32.15)	1.202	.273
III	24 (77.42)	211 (67.85)		
ISS (n, %)				
<III	13 (41.94)	162 (52.09)	1.163	.281
III	18 (58.06)	149 (47.91)		
Pathological fracture (n, %)				
No	18 (58.06)	213 (68.49)	1.397	.237
Yes	13 (41.94)	98 (31.51)		
CVC (n, %)				
No	10 (32.26)	175 (56.27)	6.545	.011
Yes	21 (67.74)	136 (43.73)		
Platelet count (×10^9^/L) (mean, SD)	160.27 ± 9.11	163.56 ± 8.69	1.926	.055
Neutrophil count (×10^9^/L) (mean, SD)	7.34 ± 1.12	7.47 ± 1.33	0.551	.582
WBC (%) (mean, SD)	7.65 ± 1.56	7.57 ± 1.62	0.294	.769
Albumin (g/L) (mean, SD)	25.04 ± 3.23	25.64 ± 3.09	0.992	.322
β2-microglobulin (mg/L) (mean, SD)	6.08 ± 1.18	6.39 ± 1.32	1.403	.162
CRP (mg/L) (mean, SD)	3.90 ± 0.53	4.74 ± 0.87	7.781	<.001
Lactate dehydrogenase (U/L) (mean, SD)	138.22 ± 10.75	134.49 ± 11.13	1.834	.067
Serum creatinine (umol/L) (mean, SD)	173.71 ± 12.23	169.23 ± 13.38	1.789	.075
Fibrinogen (g/L) (mean, SD)	4.03 ± 0.36	3.11 ± 0.58	0.8661	<.001
D-dimer (μg/mL) (mean, SD)	1.25 ± 0.25	1.04 ± 0.30	0.3693	<.001
vWF (%) (mean, SD)	152.41 ± 17.22	173.68 ± 14.18	6.654	<.001
FVIII (%) (mean, SD)	140.55 ± 18.76	155.48 ± 12.64	4.333	<.001
IMiD (n, %)				
No	18 (58.06)	110 (35.37)	6.200	.013
Yes	13 (41.94)	201 (64.63)		
Aspirin (n, %)				
No	9 (29.03)	104 (33.44)	0.248	.619
Yes	22 (70.97)	207 (66.56)		
Low molecular heparin (n, %)				
No	24 (77.42)	186 (59.81)	3.690	.055
Yes	7 (22.58)	125 (40.19)		

CRP = C-reactive protein, CVC = central venous catheter, DS = Durie–Salmon staging, FVIII = coagulation factor VIII, IMiD = immunomodulator, ISS = International staging system staging, MM = multiple myeloma, VTE = venous thromboembolic events, vWF = von Willebrand factor, WBC = white blood cell.

### 3.3. Multivariate logistic regression analysis of MM patients complicated with VTE

The 8 variables screened by univariate analysis were included in multivariate LR analysis. The assignment showed that CVC (No = 0, Yes = 1) and treatment regimen contained IMiD (No = 0, Yes = 1). Age, CRP, fibrinogen, D-dimer, vWF, and FVIII were the original input. The results show that for each 1 mg/L increase in CRP, the risk of VTE in patients increases by 7.745 times; for each 1 g/L increase in FIB levels, the risk of VTE in patients increases nearly 20.963 times; for each 1% increase in vWF, the risk of VTE in patients increases by 1.064 times; for each 1% increase in FVIII, the risk of VTE in patients increases by 1.061 times; treatment plans involving IMiD may increase the risk of VTE to 4.818 times (see Table 2).

**Table 2:** Multivariate LR analysis of MM patients complicated with VTE.

Variables	β	SE	Waldx^2^	*P*	OR	95% CI
CRP (mg/L)	2.047	0.565	13.127	<.001	7.745	2.559 to 23.441
Fibrinogen (g/L)	3.043	0.681	19.945	<.001	20.963	5.515 to 79.688
vWF (%)	0.062	0.023	7.035	.008	1.064	1.016 to 1.113
FVIII (%)	0.059	0.023	6.700	.010	1.061	1.014 to 1.110
IMiD (yes)	1.572	0.685	5.271	.022	4.818	1.259 to 18.443
Constant	−48.938	8.439	33.63	<.001	-	-

CRP = C-reactive protein, FVIII = coagulation factor VIII, IMiD = immunomodulator, LR = logistic regression, MM = multiple myeloma, VTE = venous thromboembolic events, vWF = von Willebrand factor.

**Figure 1.**
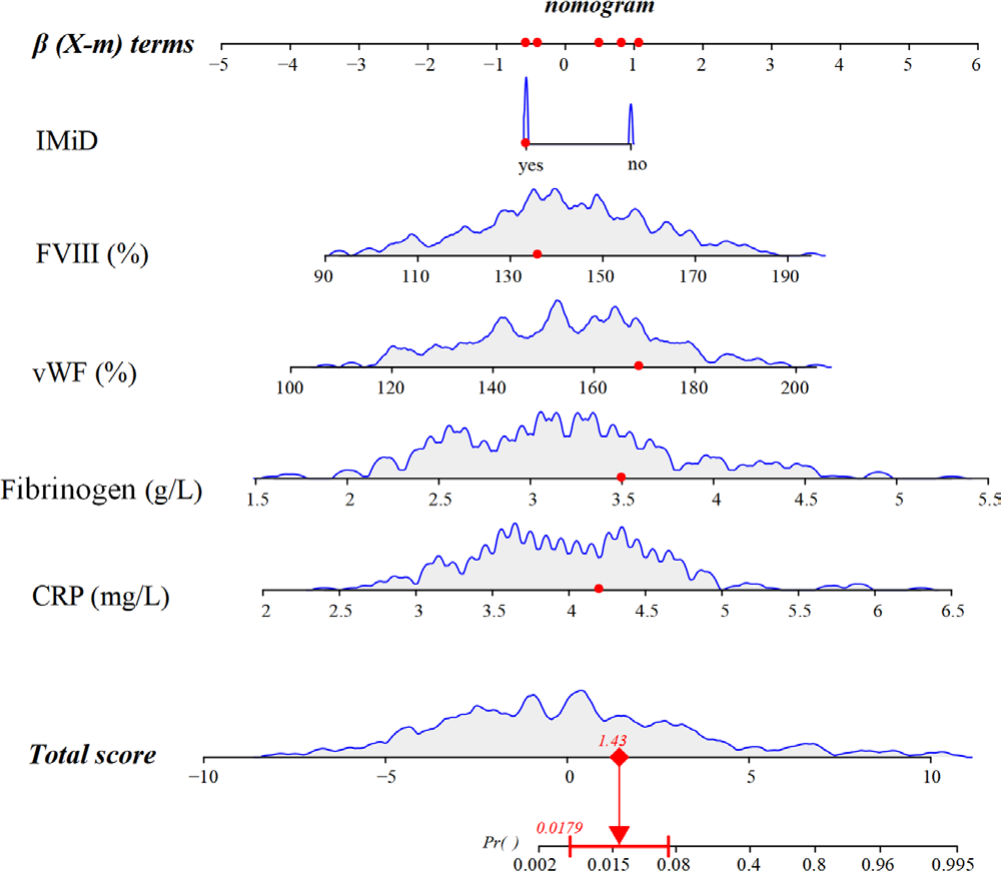
Nomogram model.

A nomogram prediction model for the occurrence of patients with VTE in MM was constructed (Fig. 1). Taking a patient as an example, the predicted probability of concurrent VTE was calculated: the patient’s treatment plan includes IMiD, with FVIII at 165%, vWF at 180%, fibrinogen at 4.2g/L, and CRP at 3.4mg/L. According to the nomogram, the predicted probability of VTE for this MM patient is 0.0179, or 1.79%.

**Figure 2.**
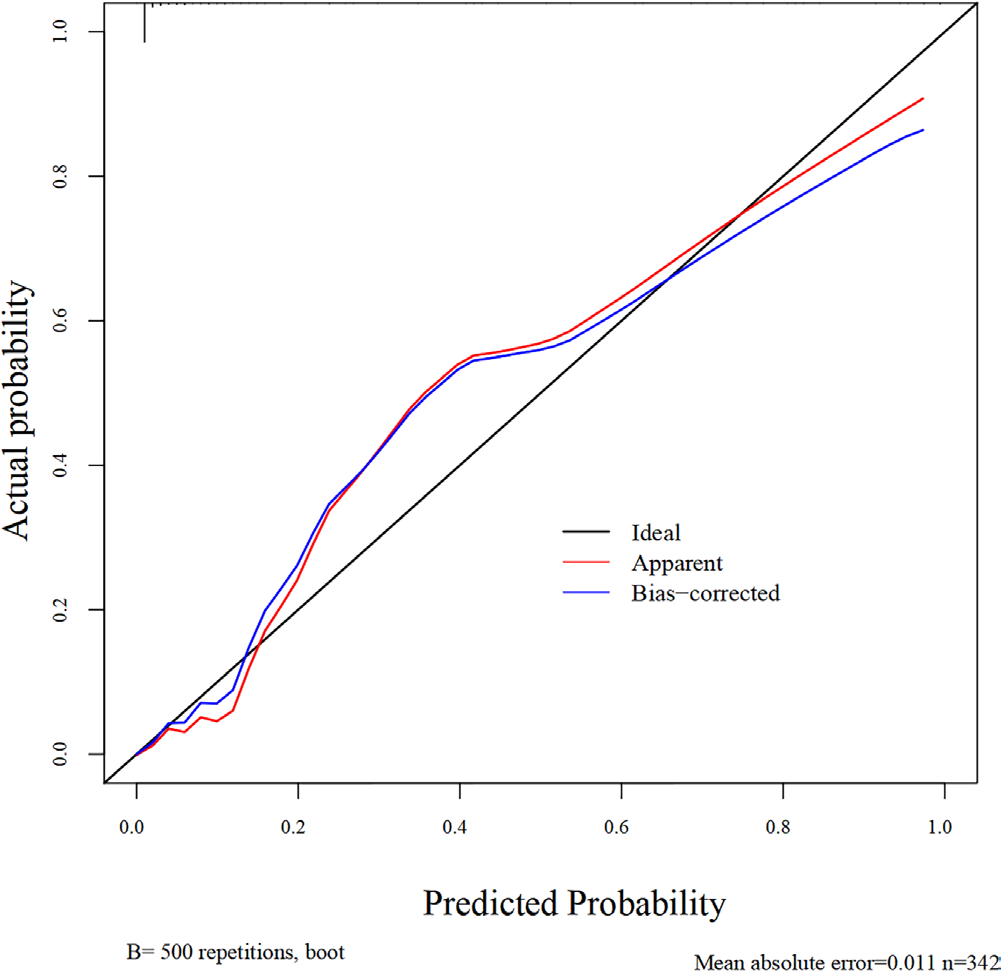
Calibration curve of the nomogram model predicting VTE in MM patients. MM = multiple myeloma, VTS = venous thromboembolic events.

We used the Bootstrap method, and through 500 resampling validations of the nomogram model, we found that the MAE of the calibration curve was 0.011, indicating a good consistency between the predicted risk and the actual occurrence risk (see Fig. 2). The goodness-of-fit bias test showed no statistically significant difference between the predicted values and the actual values (χ²=1.777, *P* = .987 > .05), suggesting that the predictive model does not exhibit overfitting.

### 3.4. Construct the RF of thrombus in MM patients

The RF model is an ensemble learning method based on decision trees. It improves the model’s accuracy by constructing multiple decision trees and combining their prediction results. A higher Gini index indicates that the variable has greater importance in the model’s predictions.

We used the RF model to assess the risk factors for VTE in patients with MM, ranking the importance of variables based on the Gini index (see Table 3). The results showed that CRP and fibrinogen were the main risk predictors, with Gini indices of 12.76 and 12.31, respectively, indicating their critical role in VTE assessment. Additionally, vWF and FVIII also exhibited high importance (Gini indices of 7.60 and 4.76, respectively), emphasizing their role in blood coagulation. The Gini index for age was 4.30, suggesting that increasing patient age is associated with a higher risk of VTE. Although other variables (such as platelet count, D-dimer, β2-microglobulin, serum creatinine, and albumin) had relatively lower importance, they still provide clinical reference value.

**Table 3:** Ranking of variable importance in RF model.

Importance ranking	Variables	Gini index
1	CRP	12.76
2	Fibrinogen	12.31
3	vWF	7.60
4	FVIII	4.76
5	Age	4.30
6	Platelet count	2.92
7	D-dimer	3.51
8	β2-microglobulin	2.65
9	Serum creatinine	3.18
10	Albumin	2.79

CRP = C-reactive protein, FVIII = coagulation factor VIII, RF = random forest, vWF = von Willebrand factor.

### 3.5. Construct the GBM of thrombus in MM patients

**Figure 3.**
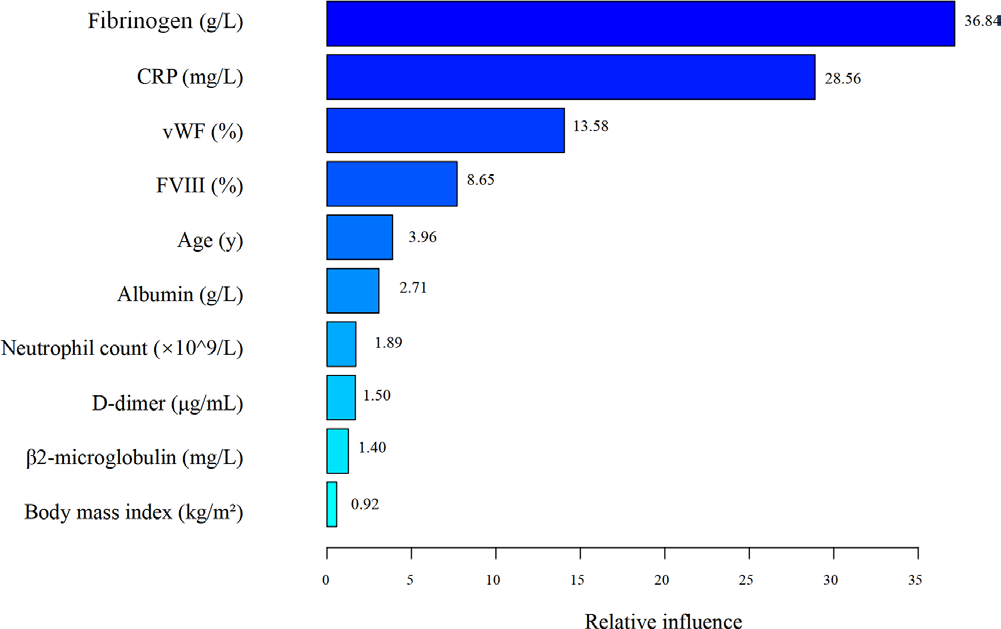
Ranking of variable importance in GBM. GBM = gradient boosting machine.

The importance was ranked based on the average relative influence of each independent variable during the model-building process. Fibrinogen and CRP were the 2 most important predictors of VTE risk, with average relative influences of 36.84 and 28.56, respectively. Additionally, vWF and FVIII ranked third and fourth, with influences of 13.58 and 8.65. Age, with an influence of 3.96, ranked fifth, suggesting that increasing age may raise the risk of VTE. Other variables such as albumin, neutrophil count, β2 microglobulin, D-dimer, and body mass index had relatively weaker predictive power for VTE risk, with influences of 2.71, 1.89, 1.50, 1.40, and 0.92, respectively (see Fig. 3).

### 3.6. Validation of prediction efficacy

**Figure 4.**
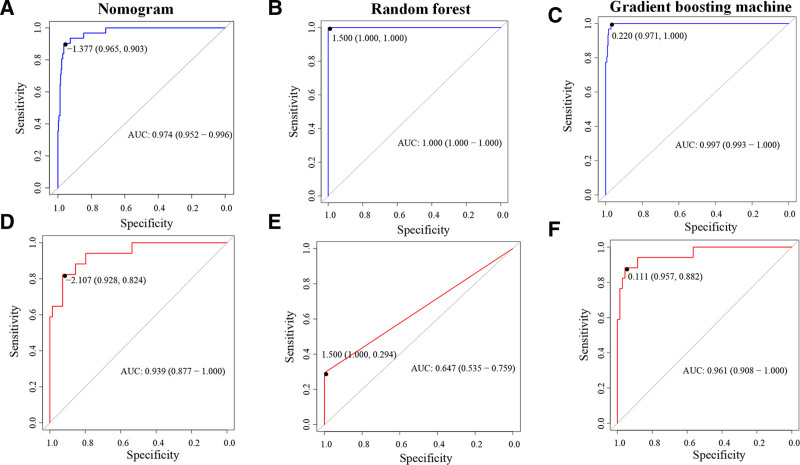
ROC curve analysis of the 3 models; (A–C) training set; (D–F) validation set. ROC = receiver operating characteristic curve.

We further compared the predictive performance of the 3 models by calculating the sensitivity, specificity, and AUC predicted by the models. Among the 3 models, the RF model showed higher sensitivity and AUC values in the training set, followed by GBM, and then the nomogram. Compared to the training set, the predictive metrics for all 3 models decreased in the validation set, with GBM showing the best predictive performance (see Fig. 4).

## 4. Discussion

There are some differences in the incidence of VTE in MM patients reported by different studies. It has been reported that the incidence of MM with thrombosis during treatment is about 10%.^[10,11,13]^ In this study, the incidence of VTE in MM patients was 11.21 %. The main type of thrombosis is lower extremity deep vein thrombosis, which is consistent with related reports.^[10,14]^ The formation of thrombosis in MM is the result of multifactorial interactions, including general factors, disease-related factors, and treatment-related factors. However, the exact mechanisms are still unclear. In MM patients, the mortality rate within 1 year after diagnosis is 3 times higher in those who develop VTE compared to those who do not.^[15]^ A large cohort study conducted by Schoen MW et al^[16]^ in 2020, involving 4446 patients, found that early VTE events (occurring 6–12 months after treatment) were associated with increased mortality in MM patients. Therefore, predicting the risk of thrombosis in MM patients is of great significance in guiding clinical treatment and preventive measures.

Several studies have investigated the risk factors for thrombosis in MM patients. Li et al^[14]^ found that a history of VTE, recent surgery within 90 days, and age ≥80 years were independent predictors of thrombosis in MM patients. Sanfilippo et al^[17]^ conducted a retrospective analysis and found that a body mass index ≥25 kg/m^2^, recent hip fracture, history of VTE, and CVC placement increased the risk of thrombosis in MM patients. However, another study involving 190 MM patients found no association between common thrombotic risk factors such as age, weight, history of heart disease, history of diabetes, surgery, and CVC placement with the occurrence of thrombosis.^[18]^ These study results suggest that the risk factors for thrombosis in MM patients may vary and may be related to the characteristics of the disease itself and individual differences in the study population.

Based on LR, RF, and GBM, we screened fibrinogen, vWF, FVIII, and CRP as predictors of VTE in MM patients. Thrombosis is a complex physiological process involving the interaction of multiple coagulation factors, platelets and vascular endothelial cells. The abnormality of coagulation function may lead to the formation of thrombus, and the increase of fibrinogen in the coagulation function index indicates that the procoagulant activity in the body is enhanced.^[19]^ There is a certain correlation between fibrinogen level and the risk of VTE in MM patients. High levels of fibrinogen may mean the hyperfunction of coagulation and increase the risk of thrombosis. Therefore, fibrinogen can be considered as a potential predictor for assessing the risk of VTE in MM patients.

The hypercoagulable state in MM patients is associated with the upregulation of coagulation factors such as tissue factor, FVIII, and vWF. Auwerda et al^[20]^ found that the levels of FVIII and vWF antigen and activity were significantly higher in MM patients compared to healthy controls, and were significantly correlated with the ISS disease stage. The elevated levels of FVIII and vWF may be a response to increased neoangiogenesis in the bone marrow microenvironment.^[21]^ In a study by Minnema et al,^[22]^ MM patients treated with thalidomide showed extremely high levels of FVIII activity (FVIII:C) and vWF antigen, with average FVIII:C levels at 352% and average vWF antigen levels at 374%. However, the relative contribution of these mechanisms in regulating the hypercoagulable state in MM patients is still not clear. Although elevated plasma vWF levels have been associated with increased risk of VTE and worse prognosis, other coagulation factors and disease characteristics may also influence thrombus formation.

The disease-related factors associated with the development of VTE in MM are related to the increased production of inflammatory factors mediated by clonal plasma cells. In this study, the inflammatory marker CRP was also found to be a predictive factor for thrombosis in MM patients. CRP is an inflammation marker commonly used to assess the degree of inflammatory response in the body. Elevated levels of CRP may reflect the inflammatory state of the disease and tumor burden,^[23]^ both of which can contribute to thrombus formation. Additionally, CRP is also associated with the activation of the coagulation system and changes in platelet function, which may also contribute to the occurrence of thrombotic events.

Multiple myeloma patients often require long-term chemotherapy or stem cell transplantation, which may involve the use of CVCs, increasing the risk of developing blood clots. In this study, an analysis was conducted on 20 patients who developed blood clots within 6 months after diagnosis, and it was found that 13 of these MM patients had clot formation related to the catheter. This suggests that catheter-related thrombosis is 1 of the main causes of delayed thrombosis in MM patients. Treatment-related factors in MM can further exacerbate the risk of VTE. Multiple studies have shown that the use of IMiDs such as thalidomide and lenalidomide can further increase the risk of VTE in MM patients.^[13,17]^ In the results of this study, the multivariate analysis indicated that the use of IMiD is a risk factor for VTE in patients with MM, but this finding was not supported by the RF model or the GBM model. The reasons for the differences between the results of this study and previous reports may be due to the small sample size and the limitations of retrospective analysis, where there are many confounding factors that cannot truly reflect the relationship between VTE and treatment factors.

Through the systematic identification of these factors, important guidance can be provided for doctors in clinical practice, helping them better identify and manage high-risk patients with MM. Firstly, doctors can classify MM patients based on the levels of these biomarkers, clearly identifying which patients are in a high-risk state and implementing stricter monitoring and management for this group. For example, for patients with significantly elevated levels of FIB and vWF, consideration can be given to increasing the use of anticoagulant medications in their treatment plan, which will help reduce the incidence of VTE. Secondly, by comprehensively considering the clinical background and biomarker levels of patients, doctors can optimize treatment strategies, selecting appropriate anticoagulant measures and making dynamic adjustments. By continuously monitoring the levels of CRP and FVIII, doctors can promptly identify potential changes in risk and make corresponding adjustments to safeguard the health and safety of patients. Finally, the findings of this study not only enhance our understanding of the risk factors for VTE in MM patients but also provide a scientific basis for improving clinical pathways. In the future, with the accumulation of more data and optimization of models, personalized prevention strategies based on biomarkers are expected to be more widely applied.

In the study, the limitation of sample size may significantly impact the stability, predictive accuracy, and generalizability of the models. Specifically, insufficient sample size can lead to overfitting in regression analysis, particularly when there are many variables involved. This makes the model overly sensitive to noise and outliers in the training data, resulting in reduced performance on new data. Additionally, a smaller sample size may also lead to instability in the estimation of model parameters, especially when dealing with high-dimensional data, where issues of bias-variance trade-off can arise and potentially affect the reliability of the research conclusions.

In this study, the 3 models we used LR-based nomogram, RF, and GBM – are designed to maintain high predictive performance and reliability in small sample data through appropriate methods and ensemble approaches. A nomogram prediction model is constructed based on the results of LR. The nomogram visually presents the contribution of each variable to the outcome based on the regression coefficients, enhancing the interpretability of the model. To avoid overfitting in the case of small sample sizes, we employed cross-validation to evaluate the model’s generalizability. Cross-validation effectively assesses the model’s performance across different data splits, reducing biases that may arise from data partitioning. Additionally, the bootstrap method was used to estimate the stability and uncertainty of the model parameters, further strengthening the robustness of the nomogram. This method generates multiple different training samples (e.g., by random sampling with replacement) to estimate the model’s prediction error, ensuring the consistency of regression coefficients across various data subsets.

RF is particularly effective in handling small sample data. It uses the Bootstrap method to randomly sample training data and randomly selects subsets of features during the construction of each decision tree, thereby reducing the model’s reliance on any particular sample or feature. This built-in randomness allows RF to effectively avoid overfitting, providing stable and relatively accurate predictions even with a small sample size. GBM achieves good predictive performance on small sample data by progressively reducing prediction errors. GBM adjusts the bias of the model in each iteration, making the model more sensitive to the errors from the previous round. This process improves the model’s accuracy, and especially in cases with limited data, hyperparameters (such as learning rate, tree depth, etc.) can be adjusted to prevent overfitting.

Although our study has the limitation of a small sample size, we have made efforts to minimize the potential bias associated with small samples through appropriate model selection and methods such as cross-validation. We recognize that a small sample may impact the stability and generalizability of regression models; therefore, we emphasize that the results of this study should be validated in research with a larger sample size.

## 5. Limitations

Nevertheless, there are still some shortcomings in this study: study was small sample sized. Some factors may not be taken into account, or there are measurement errors. The results of the study need to be verified by external data sets to determine its stability and scope of application.

## 6. Conclusion

Overall, the analysis of risk factors for thrombosis in MM is a complex process that requires the comprehensive use of various predictive models. By utilizing advanced algorithms such as LR, RF, and GBM, we effectively identified fibrinogen, vWF, FVIII, and CRP as predictive factors for venous thromboembolism in patients with MM. These findings not only provide a scientific basis for high-risk screening in MM patients but also offer practical approaches for clinicians to identify and manage high-risk patients in their practice. As research advances, future clinical management will become more precise, contributing to the improvement of survival quality and safety for MM patients.

## Author contributions

**Conceptualization:** Yi Huang, Haimei Liang.

**Data curation:** Yi Huang, Haimei Liang.

**Formal analysis:** Yi Huang, Haimei Liang.

**Funding acquisition:** Yi Huang.

**Investigation:** Yi Huang, Haimei Liang, Shaoxin Huang, Xueli Xie.

**Methodology:** Yi Huang, Haimei Liang, Shaoxin Huang, Xueli Xie.

**Resources:** Yi Huang.

**Software:** Shaoxin Huang, Xueli Xie, Bin Deng.

**Visualization:** Yi Huang, Haimei Liang, Shaoxin Huang, Wenjie Liang.

**Writing – original draft:** Yi Huang.

**Writing – review & editing:** Yi Huang.
